# Association between endometriosis and cardiovascular disease: a population-based cohort study

**DOI:** 10.7189/jogh.16.04326

**Published:** 2026-09-18

**Authors:** Xueyin Wang, Shuting Xie, Huan Yu, Kun Wang, Huairong Wang, Ruotong Yang, Xueying Qin, Yiqun Wu, Yonghua Hu

**Affiliations:** 1 Department of Obstetrics and Gynecology and Reproductive Medicine, Peking University First Hospital, Beijing, China; 2 Department of Epidemiology and Biostatistics, School of Public Health, Peking University Health Science Center, Beijing, China; 3 Key Laboratory of Epidemiology of Major Diseases (Peking University), Ministry of Education, Beijing, China

**Keywords:** endometriosis, cardiovascular disease, cerebrovascular disease, population-based cohort study, epidemiology

## Abstract

**Background:**

The relationship between endometriosis and cardiovascular disease (CVD) risk remains unclear. This study aimed to evaluate the association between endometriosis and CVD risk, to examine whether the association was affected by age group, and to exploratorily assess the mediating role of medications in these associations.

**Methods:**

A historical, population-based cohort study was conducted based on the Beijing Medical Claim Data for Employees between 2010 and 2017. A total of 19,903 women with endometriosis and 79,612 controls were identified by matching age and year of endometriosis diagnosis in a 1:4 ratio. The primary outcome was the incident diagnosis of any CVD events, and secondary outcomes were major CVD and cerebrovascular disease. The hazard ratios (HRs) were estimated by Cox proportional hazards models representing the association between endometriosis and CVD.

**Results:**

Endometriosis was associated with an increased risk of CVD after adjusting for urbanisation, hypertension and diabetes mellitus (HR = 1.69; 95% CI = 1.35–2.12). The adjusted HRs for major CVD and cerebrovascular disease in women with endometriosis were 1.83 (95% CI = 1.32–2.55) and 1.57 (95% CI = 1.12–2.22) compared with controls, respectively. Interaction analyses between endometriosis and age group (18–44 years *vs.* 45–55 years) yielded *P*-values of 0.942, 0.552, and 0.211 for all CVD, major CVD, and cerebrovascular disease, respectively, indicating no statistically significant heterogeneity in the associations across age groups. In the overall population, a proportion of the association between endometriosis and all CVD risk was statistically mediated by statins (68.30%) and aspirin (78.15%).

**Conclusions:**

Endometriosis was associated with an increased risk of CVD.

Cardiovascular disease (CVD) is the leading cause of death and disease burden worldwide, and it has been estimated that 523 million people suffered from CVD, and 18.6 million people died of CVD globally in 2019 [1]. In China, CVD remains the main cause of both mortality and premature mortality, and accounts for 40% of deaths [2]. Evidence has shown that the incidence and mortality of CVD fell steeply in individuals aged ≥55 years, whereas populations below 55 years experienced an increase in CVD incidence and a small decrease in CVD mortality, particularly in female populations [3,4]. Although several female sex-specific contributors to CVD including polycystic ovary syndrome, adverse pregnancy outcomes, and premature menopause have been identified [5–7], other risk factors specific to younger women including endometriosis are still unclear and needed to be explored to further reduce CVD burdens in this population.

Endometriosis is an oestrogen-dependent chronic gynaecologic disease that has been estimated to affect 10% of reproductive-age women [8]. It is characterised by the presence of endometrium-like tissue outside the uterus and may lead to several symptoms and disorders including pelvic pain, dysmenorrhea, and infertility [9,10]. Endometriosis has been correlated with systemic chronic inflammation, atherogenic lipid profile, and elevated oxidative stress, which also contribute to the formation and progression of coronary artery atherosclerosis and an increased risk of CVD [11,12]. Several population-based cohort studies have indicated an association between endometriosis and CVD risk [13–15]. The Nurses’ Health Study II of 116,430 US women suggested an association of laparoscopically confirmed endometriosis with a higher risk of coronary heart disease [13]. Another population-based retrospective cohort study of 279,759 UK women reported a 1.24-fold increased risk for composite CVD in women with endometriosis compared with those without endometriosis [14]. Recently, a cohort study of 500,559 Canadian women found a 14% increased risk for hospital admission for CVD and a 26% greater risk for secondary CVD events in women with endometriosis [15].

Previous studies on the relationship between endometriosis and CVD risk primarily focused on Western populations, and only findings from Taiwan’s National Health Insurance Research Database suggested a significant association between endometriosis and the risk of CVD events in Asian women [16]. Moreover, age has been considered to modify the link between endometriosis and CVD risk with inconsistent results. Findings from the study by Mu *et al*. [13] indicated a decrease in the relative risk for coronary heart disease events as age increased, while the research by Blom *et al*. [15] only observed the increased risk of CVD in women with endometriosis among women aged below 45 years. In addition, previous evidence demonstrated that medical treatment for endometriosis was associated with a 30% reduced risk of major adverse cardiovascular and cerebrovascular events [16], whereas hormone therapy was reported to mediate 31.0% and 15.5% of the association between endometriosis and coronary heart disease and stroke, respectively [13,17]. Prior studies among Taiwanese women have investigated the association between endometriosis and CVD and reported that women who used aspirin or statins had a significantly increased risk of coronary artery disease compared with non-users [18,19]. However, the effect of medications used to treat or manage endometriosis and prevent CVD on the associations remains unclear.

Therefore, a better understanding of the relationship between endometriosis and CVD risk among Asian females are necessary. This study aimed to evaluate the association between endometriosis and CVD risk in Chinese women, and examine whether the association was affected by age group, and further exploratorily assess the mediating role of medications in these associations.

## METHODS

### Study source and study population

This study adhered to Journal of Global Health’s GRABDROP [20] and compliance with these guidelines is detailed in Table S1 in the **Online Supplementary Document**. We conducted a historical, population-based matched cohort study using data from the Beijing Medical Claim Data for Employees (BMCDE) between 2010 and 2017. The BMCDE includes clinical diagnosis, medications, and medical claim information for working or retired urban residents enrolled in the Urban Employee Basic Medical Insurance (UEBMI) program in Beijing, China. The UEBMI is one of three basic medical insurances in China covering more than 80% of residents in Beijing [21]. All primary, secondary, and tertiary hospitals are centralised in the UEBMI system; thus, each hospitalisation is recorded. The BMCDE database has been previously introduced in detail elsewhere [21]. This study was exempted from institutional review board approval by the ethics committee of Peking University Health Science Center.

We identified women aged 18–55 years with a new diagnosis of endometriosis between 1 January 2010 and 31 December 2017. Patients with endometriosis were identified from the BMCDE database by the following inclusion criteria:

• with ≥1 in-hospital diagnosis or hospital admission for endometriosis (International Classification of Diseases 10th version (ICD-10) code of N80) between 1 January 2010 and 31 December 2017, with two-year wash-out period,

• aged 18–55 years. 

The index date was defined as the first diagnosis or hospital admission for endometriosis. Patients with a diagnosis of CVD before the index date were excluded. All patients were matched to four women with no diagnosis of endometriosis by age (±1 year), and year of endometriosis diagnosis (±1 year) who were randomly selected from the BMCDE database.

### Follow-up period

The incident CVD events were observed from the index date to the exit date. The exit date was the earliest of the outcome, study end date (31 December 2017), or death.

### Outcomes

The primary outcome was the incident diagnosis of any CVD events (ICD-10 codes I00-I99). Secondary outcomes were major CVD (including acute myocardial infarction (ICD-10 code I21), ischemic heart disease (ICD-10 code I20-I25), and heart failure (ICD-10 code I50)), and cerebrovascular disease (including stroke (ICD-10 codes I60-I64) and other cerebrovascular diseases (ICD-10 codes I65-I69)).

### Covariates and medications

Covariates included urbanisation, hypertension, and diabetes mellitus. They were acquired at the index date. Hypertension was defined as having been diagnosed with an ICD-10 code of I10-I15 or having anti-hypertensive prescriptions. Diabetes mellitus was defined as having been diagnosed with an ICD-10 code of E11-E14 or having ≥1 prescription of hypoglycaemic agents. Medications included progesterone, gonadotropin-releasing hormone (GnRH) agonists, statins, and aspirin.

### Statistical analysis

Baseline characteristics are presented as mean ± SD (standard deviation) or median (interquartile range, IQR) for continuous variables and number (percentage) for categorical variables and compared between the two groups. The incidence rate of CVD and its subtypes was calculated and showed as 95% confidence intervals (CIs) per 10,000 person-years for each group. Univariate and multivariable weighted Cox proportional hazard regression models were conducted to estimate crude and adjusted hazard ratios (HRs) and 95% CIs for the presence of endometriosis [22]. We adjusted for urbanisation, hypertension and diabetes mellitus in the multivariable models. In addition, we further conducted stratified analysis by age groups (18–44 years and 45–55 years). Mediation analyses were used to examine the proportional contribution of medications on the associations of endometriosis with the risk of CVD.

Since hypertension, diabetes mellitus and use of medications (progesterone, GnRH agonists, statins, and aspirin) has been reported to be associated with CVD risk, several sensitivity analyses for overall CVD were performed restricting analysis to:

• women without hypertension

• women without diabetes mellitus

• women without a current prescription for progesterone

• women without a current prescription for GnRH agonists

• women without a current prescription for statins

• women without a current prescription for aspirin.

All *P*-values are two-sided, and a 0.05 level was used to declare significant differences. All analyses were performed using the SAS, version 9.4 (SAS Institute Inc., North Carolina, USA).

## RESULTS

### Characteristics of study participants

**Figure 1:**
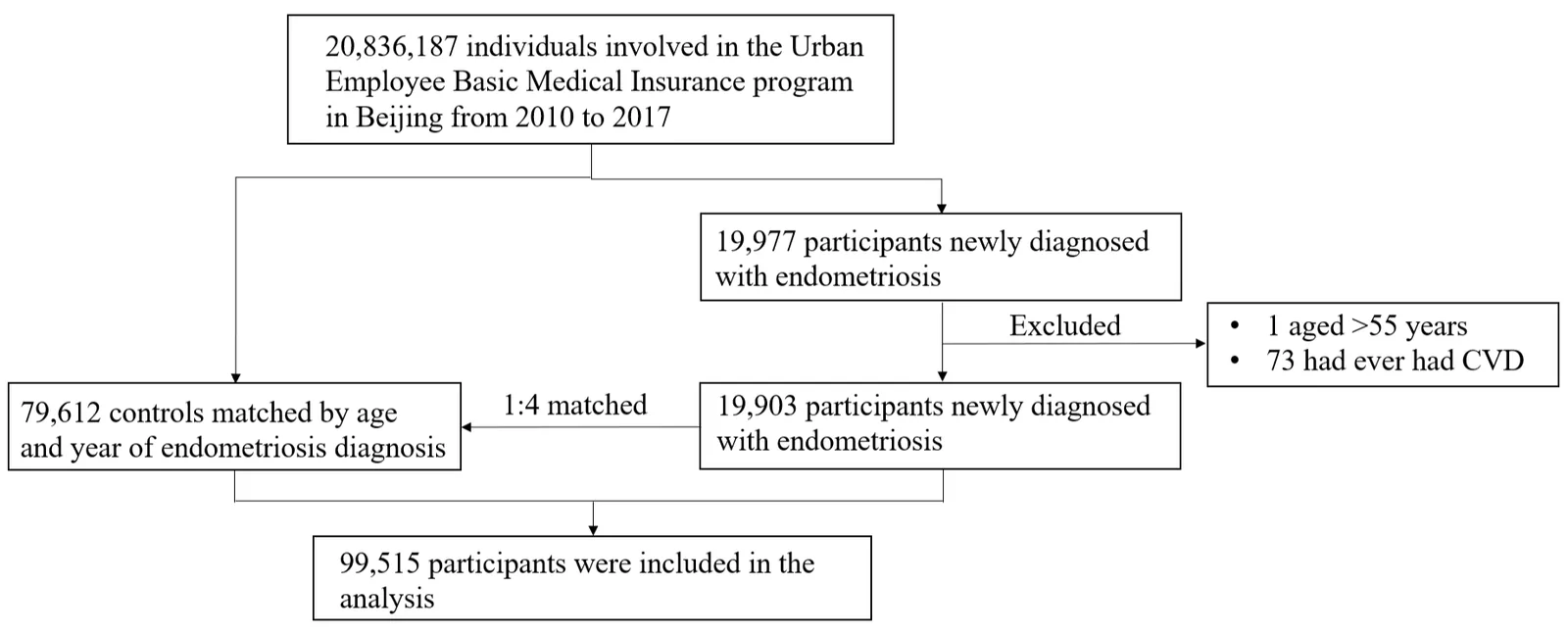
Flowchart of study cohort.

A total of 99,515 women were included in this analysis, composed of 19,903 women newly diagnosed with endometriosis and 79,612 women without endometriosis (**Figure 1**). The average age at the index date was 38.4 years, 76.0% residing in urban areas, 9.5% diagnosed with hypertension, and 5.0% diagnosed with diabetes mellitus at baseline. Women with endometriosis were more likely to reside in urban areas, and have higher incidence of hypertension and diabetes mellitus (all *P* < 0.001). Women with endometriosis were more likely to have a current prescription for statins, aspirin, progesterone, and GnRH agonists (all *P* < 0.001). The baseline characteristics of study participants with endometriosis and matched controls without a diagnosis of endometriosis have been shown in **Table 1**.

**Table 1:** Baseline characteristics of participants with endometriosis and without endometriosis

Characteristics	Total (n = 99,515)	Endometriosis (n = 19,903)	Unexposed (n = 79,612)	*P-*value
Age in years, mean ± SD	38.4 ± 8.2	38.4 ± 8.2	38.4 ± 8.2	-
Age in years, n (%)				-
*<45 years*	73,420 (73.8)	14,684 (73.8)	58,736 (73.8)	
*≥45 years*	26,095 (26.2)	5219 (26.2)	20,876 (26.2)	
Urbanisation, n (%)	75,617 (76.0)	16,177 (81.3)	59,440 (74.7)	<0.001
Hypertension, n (%)	9450 (9.5)	2521 (12.7)	6929 (8.7)	<0.001
Diabetes mellitus, n (%)	4953 (5.0)	1350 (6.8)	3603 (4.5)	<0.001
Statins, n (%)	13,557 (13.6)	4038 (20.3)	9519 (12.0)	<0.001
Aspirin, n (%)	13,066 (13.1)	4028 (20.2)	9038 (11.4)	<0.001
Progesterone, n (%)	4339 (4.4)	2362 (11.9)	1977 (2.5)	<0.001
GnRH agonists, n (%)	3880 (3.9)	3720 (18.7)	160 (0.2)	<0.001

GnRH – Gonadotropin-releasing hormone, SD – standard deviation

### Incidence of CVD

With a median follow-up of 3.6 (IQR = 1.7–5.5) years among 365,011.3 person-years, 123 and 288 incident CVD events were recorded in women with and without endometriosis, respectively (**Table 2**). The incidence rate of all CVD in women with endometriosis was 168.8 (95% CI = 143.3–194.3) per 100,000 person-years, and 98.6 (95% CI = 79.1–118.1) per 100,000 person-years in women without endometriosis.

**Table 2:** Incidence rates and hazard ratios for cardiovascular disease events

Outcomes	Endometriosis (n = 19,903)			Unexposed (n = 79,612)			Crude HR (95% CI)	*P*-value	Adjusted HR (95% CI)†	*P*-value
	**Events, n**	**Follow-up duration in years, Mdn (IQR)**	Incidence rate (95% CI)/100,000 person-years*	**Events, n**	**Follow-up duration in years, Mdn (IQR)**	Incidence rate (95%CI)/100,000 person-years*				
All CVD	123	3.6 (1.7–5.5)	168.8 (143.3–194.3)	288	3.6 (1.6–5.5)	98.6 (79.1–118.1)	1.71 (1.39–2.11)	<0.001	1.69 (1.35–2.12)	<0.001
Major CVD	54	3.6 (1.7–5.5)	74.1 (57.2–91.0)	124	3.6 (1.6–5.5)	42.4 (29.6–55.2)	1.77 (1.28–2.44)	0.013	1.83 (1.32–2.55)	<0.001
Cerebrovascular disease	55	3.6 (1.7–5.5)	75.5 (58.5–92.5)	137	3.6 (1.6–5.5)	46.9 (33.5–60.3)	1.59 (1.16–2.17)	0.004	1.57 (1.12–2.22)	0.010

CI – confidence interval, CVD – cardiovascular disease, HR – hazard ratio, IQR – interquartile range, Mdn – median

*Person-years for endometriosis and control groups were 72,873.8 and 29,2137.5, respectively.

†Adjusted for urbanisation, hypertension, and diabetes mellitus.

There were 54 and 124 incident major CVD events among women with and without endometriosis, respectively (**Table 2**). The incidence rate of major CVD in women with endometriosis was 74.1 (95% CI = 57.2–91.0) per 100,000 person-years, and 42.4 (95% CI = 29.6–55.2) per 100,000 person-years in women without endometriosis.

There were 55 and 137 incident cerebrovascular disease events among women with and without endometriosis, respectively (**Table 2**). The incidence rate of cerebrovascular disease in women with endometriosis was 75.5 (95% CI = 58.5–92.5) per 100,000 person-years, and 46.9 (95% CI = 33.5–60.3) per 100,000 person-years in women without endometriosis.

### Association between endometriosis and CVD risk

**Figure 2:**
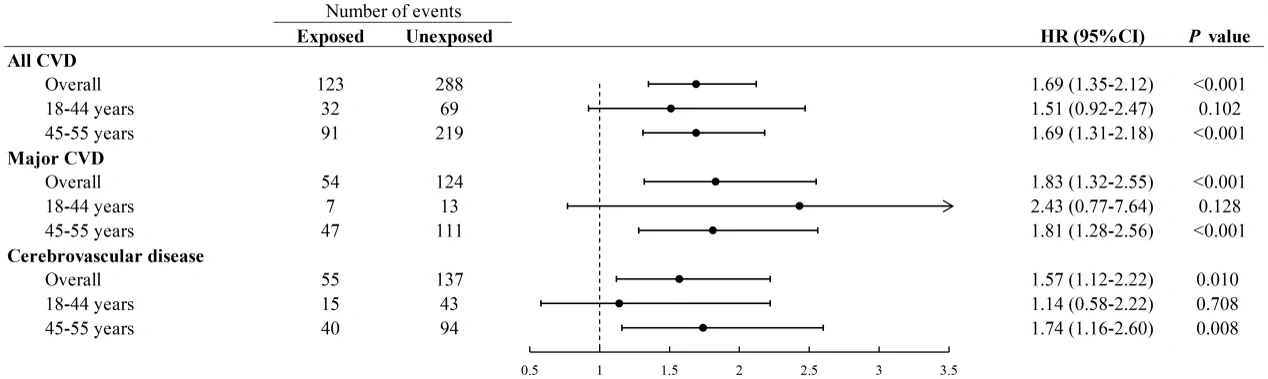
Hazard ratios (95% confidence intervals) for cardiovascular disease events. Adjusted for urbanisation, hypertension, and diabetes mellitus. CI – confidence interval, CVD – cardiovascular disease, HR – hazard ratio.

The association between endometriosis and the risk of CVD and its subtypes is presented in **Table 2** and **Figure 2**. The crude HR of all CVD among women with endometriosis compared to those without endometriosis was 1.71 (95% CI = 1.39–2.11, *P* < 0.001). After adjusting for urbanisation, hypertension and diabetes mellitus, endometriosis was also associated with an increased risk of all CVD (adjusted HR = 1.69; 95% CI = 1.35–2.12, *P* < 0.001).

On the analysis of CVD subtypes, the crude HR of major CVD was 1.77 (95% CI = 1.28–2.44, *P* = 0.013), and this association remained significant in the adjusted model (adjusted HR = 1.83; 95% CI = 1.32–2.55, *P* < 0.001). Comparing to women without endometriosis, women with endometriosis were associated with 59% (HR = 1.59; 95% CI = 1.16–2.17, *P* = 0.004) and 57% (adjusted HR = 1.57; 95% CI = 1.12–2.22, *P* = 0.010) increased risk of cerebrovascular disease in the unadjusted and adjusted model, respectively.

### Association between endometriosis and CVD risk by age group

We subsequently performed stratified analyses by age groups, 18–44 years and 45–55 years (**Figure 2**; Table S2 in the **Online Supplementary Document**). Among women aged 18–44 years, endometriosis was associated with a higher risk of all CVD in the unadjusted model (HR = 1.88; 95% CI = 1.23–2.86, *P* = 0.003), and the association was nonsignificant after adjusting for urbanisation, hypertension and diabetes mellitus (adjusted HR = 1.51; 95% CI = 0.92–2.47, *P* = 0.102). Among women aged 45–55 years, the significant associations of endometriosis with an elevated risk of all CVD were found in both unadjusted (HR = 1.66; 95% CI = 1.30–2.12, *P* < 0.001) and adjusted models (HR = 1.69; 95% CI = 1.31–2.18, *P* < 0.001).

For individual subtypes, no significant associations were observed in women aged 18–44 years for either major CVD disease in unadjusted (HR = 2.30; 95% CI = 0.90–5.84, *P* = 0.081) and adjusted (HR = 2.43; 95% CI = 0.77–7.64, *P* = 0.128) models or cerebrovascular disease for unadjusted (HR = 1.40; 95% CI = 0.78–2.51, *P* = 0.267) and adjusted (HR = 1.14; 95% CI = 0.58–2.22, *P* = 0.708) models. However, in women aged 45–55 years, endometriosis was significantly associated with an increased risk of both major CVD for unadjusted (HR = 1.71; 95% CI = 1.22–2.41, *P* = 0.002) and adjusted (HR = 1.81; 95% CI = 1.28–2.56, *P* < 0.001) models and cerebrovascular disease for unadjusted (HR = 1.67; 95% CI = 1.15–2.42, *P* = 0.007) and adjusted (HR = 1.74; 95% CI = 1.16–2.60, *P* = 0.008) models.

Interaction analyses yielded *P*-values of 0.942, 0.552, and 0.211 for all CVD, major CVD, and cerebrovascular disease, respectively, indicating no significant heterogeneity in the associations across age groups (Table S2 in the **Online Supplementary Document**).

### Exploratory mediation analyses

**Figure 3:**
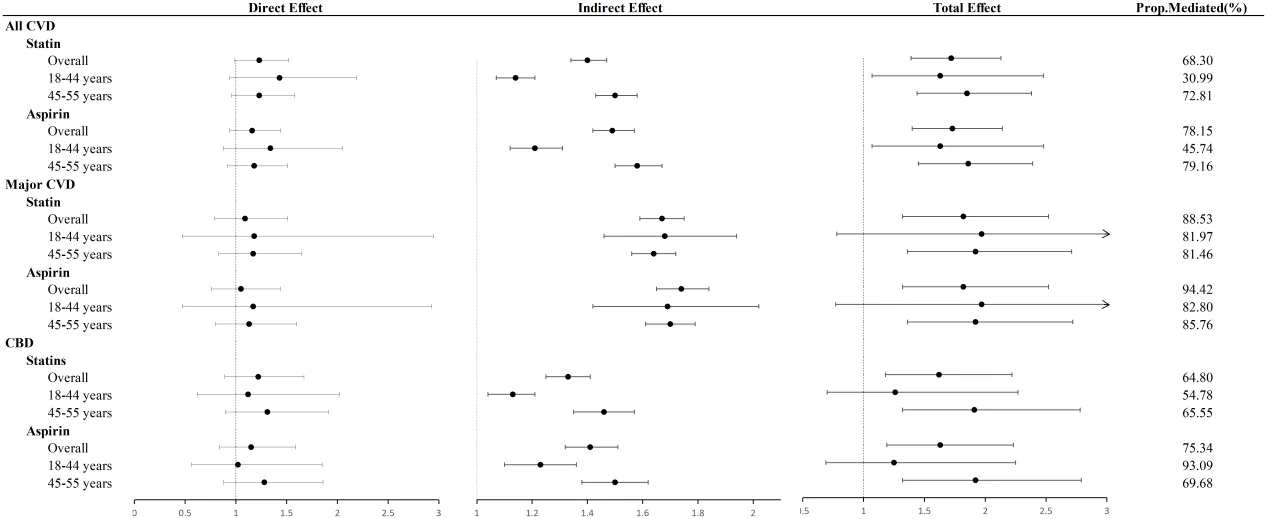
Mediation analysis to assess whether statins or aspirin mediated the associations between endometriosis and cardiovascular disease risk. Values were adjusted for urbanization, hypertension, and diabetes mellitus. Mediation proportions are reported only for descriptive purposes. Formal interpretation of the proportion mediated is warranted only when both the indirect effect and the total effect are statistically significant. Estimates exceeding 100% or negative values occur when the indirect and total effects are not consistently significant, and such estimates should not be interpreted as meaningful effect sizes. CI – confidence interval, CBD – cerebrovascular disease, CVD – cardiovascular disease, HR – hazard ratio, Prop. – proportion.

Exploratory mediation analyses for medications are presented in **Figure 3** and Table S3 in the **Online Supplementary Document**. In the overall population, we observed that a proportion of the association between endometriosis and all CVD risk was statistically mediated by statins (68.30%) and aspirin (78.15%). In age-stratified analyses, the proportions mediated by statins and aspirin appeared greater in women aged 45–55 years than in those aged 18–44 years (statins = 72.81% *vs.* 30.99%; aspirin = 79.16% *vs.* 45.74%). For major CVD, the proportions mediated by statins and aspirin were 88.53% and 94.42%, respectively, in the overall population. For cerebrovascular disease, the proportions mediated by statins and aspirin were 64.80% and 75.34%, respectively, in the overall population. Given the exploratory nature of these mediation analyses and the potential for confounding by indication, these results should be interpreted with caution.

### Sensitivity analyses

Sensitivity analyses were performed based on different restricted analyses on the association between endometriosis and CVD risk, and the results were generally consistent with those in the whole population. Specifically, the respective exclusion of women with hypertension (HR = 1.92; 95% CI = 1.44–2.56, *P* < 0.001), diabetes mellitus (HR = 1.94; 95% CI = 1.49–2.53, *P* < 0.001), and a prescription for GnRH agonists (HR = 1.73; 95% CI = 1.38–2.15, *P* < 0.001) and statins (HR = 1.86; 95% CI = 1.08–3.22, *P* = 0.024) strengthened the association between endometriosis and CVD risk, while the respective exclusion of women with a prescription for progesterone (HR = 1.65; 95% CI = 1.31–2.08, *P* < 0.001) and aspirin (HR = 1.41; 95% CI = 0.77–2.57, *P* = 0.268) resulted in a minor decrease in the effect estimate for CVD.

## DISCUSSION

In this population-based cohort study of 99,515 Chinese women aged 18–55 years, we found that endometriosis was associated with an increased risk of overall CVD, major CVD, and cerebrovascular disease independent of urbanisation, hypertension and diabetes mellitus. Interaction analyses between endometriosis and age group (18–44 years *vs.* 45–55 years) indicated no statistically significant heterogeneity in the associations across age groups. In the overall population, a proportion of the association between endometriosis and all CVD risk was statistically mediated by statins (68.30%) and aspirin (78.15%).

One important finding of this study is that endometriosis was associated with increased risk of overall CVD. This is consistent with a previous population-based cohort study of UK women that reported increased risk of composite CVD for women with endometriosis compared with those without endometriosis [14]. Recently, another population-based cohort study based on Ontario residents also indicated that women with endometriosis had a higher risk of hospital admission for CVD events [15]. In addition, a previous study of Taiwanese women reported the association between endometriosis and an increased risk of major CVD [16]. Furthermore, in line with the findings from the studies by Okoth *et al*. [14] and Chiang *et al*. [16], this study also suggested that the exclusion of women with a current prescription of GnRH agonists did not change the significant association between endometriosis and CVD risk.

Another finding of our study is that we found an association between endometriosis and an increased risk of major CVD (including acute myocardial infarction, ischemic heart disease, and heart failure) and cerebrovascular disease. In line with our findings, the study by Okoth *et al*. [14] also found an elevated risk of ischemic heart disease and cerebrovascular disease among UK women compared with controls. Our findings are also supported by a recent population-based cohort study by Chiang *et al*. [16], which suggested an increased risk of cerebrovascular events among Taiwanese women. In addition, the study of Ontario women agreed with our results suggesting an association between endometriosis and an increased risk of ischemic heart disease and cerebrovascular disease [15]. Moreover, the Nurses’ Health Study II by Mu *et al*. [13] also reported higher risk of myocardial infarction in women with endometriosis compared with those without endometriosis. Notably, caution is warranted when comparing our findings with those from the United Kingdom, the United States, Canada, and Taiwan, since regional differences in healthcare systems, diagnostic practices, treatment strategies, and baseline cardiovascular risk profiles of different regions may introduce heterogeneity across studies.

The potential biological mechanisms underlying the link between endometriosis and an increased risk of CVD remain speculative but may involve systemic chronic inflammation, elevated oxidative stress, atherogenic lipid profiles, and genetic susceptibilities. First, increasing evidence suggested that elevated levels of inflammatory factors are observed in both peripheral blood and peritoneal fluid of patients with endometriosis compared with unaffected counterparts [23,24]. Systemic chronic inflammation has been demonstrated to participate in vascular insults and atheromatous change, which may contribute to an increased cardiovascular risk among women with endometriosis. In addition, women with endometriosis have been reported to have higher levels of biomarkers of oxidative stress as well as lower levels of antioxidants, which may promote the development of atherogenesis through signalling pathways causing vascular inflammation [11,25]. Moreover, increased serum levels of low-density lipoprotein and decreased levels of high-density lipoprotein have been found among patients with endometriosis [26], which may further lead to atherosclerosis formation and progression. Furthermore, genetic susceptibilities have also demonstrated to be involved in the relationship between endometriosis and CVD risk. For example, previous genome-wide association studies identified several variants on chromosome 9p21 that were not only associated with endometriosis in females of European and Japanese ancestry [27], but also correlated with the risk of myocardial infarction, coronary artery disease and coronary heart disease in Western populations [28–30].

In addition, our findings suggest a potential mediating role of statins and aspirin in the association between endometriosis and CVD, though these results should be interpreted as exploratory and hypothesis-generating rather than as a causal pathway. Previous studies of Taiwanese women examining the association between endometriosis and CVD also reported that aspirin use was associated with an elevated risk of coronary artery disease among endometriosis patients [18], and that women who took aspirin or statins had greater odds of coronary artery disease in comparison with those who did not [19], which are partly consistent with our overall population findings. From a clinical perspective, statins are widely used to prevent CVD-related morbidity and mortality due to their positive influences on lipid profiles, and anti-inflammatory and other plaque-stabilisation roles [31], while aspirin is recommended for the primary prevention of CVD in high-risk populations by several guidelines because of its role in reducing platelet aggregation and vasoconstriction [32]. However, it is important to note that both medications are typically initiated only after cardiovascular risk factors, such as dyslipidaemia, hypertension and diabetes mellitus, have already been clinically identified. Therefore, the observed mediation pattern may partly reflect the presence of these underlying risk factors rather than a direct pharmacological effect of the medications. This implies that women receiving statins or aspirin were more likely already to have CVD risk factors, which could contribute to their greater subsequent CVD incidence. Overall, our findings are hypothesis-generating rather than causal, and suggest a plausible indirect pathway that warrants confirmation in future studies with longitudinal biomarker measurements and more detailed prescribing data.

Furthermore, no significant heterogeneity by age group was observed in our study, suggesting that both younger and older women with endometriosis may benefit from CVD prevention to reduce their long-term cardiovascular risk and associated healthcare burden. Our findings are partially consistent with data from the Taiwan National Health Insurance program, which also reported a significant association between endometriosis and major adverse cardiovascular and cerebrovascular events in women aged 35–50 years [16]. Moreover, the Nurses' Health Study demonstrated that the relative risk for coronary heart disease, comparing women with and without endometriosis, was highest among those aged ≤40 years and reduced with increasing age, although a significant association persisted in the 40–55 years group [13]. However, these prior studies mainly relied on direct comparisons of subgroup estimates rather than formal interaction testing. In contrast, our study advances the previous evidence by performing formal interaction analyses, which found the homogeneity of the association across age groups and reduced the risk of overinterpreting chance variations. Nonetheless, direct comparisons across studies are challenging due to differences in ethnicity, age categorisation, outcome definitions, and covariate adjustment strategies. Further prospective studies with larger sample sizes and extended follow-up duration, particularly in Asian populations, are warranted to confirm our findings.

To the best of our knowledge, this is the first population-based cohort study with a large sample size examining the association between endometriosis and CVD risk based on the mainland Chinese women, and used the BMCDE database that is representative of the Beijing urban population. This study has several limitations to be noted. First, the two-year washout period was a practical compromise to balance exposure accuracy and adequate follow-up, but it may not fully exclude undiagnosed prevalent cases due to diagnostic delay. Additionally, the median follow-up of 3.6 years is relatively short for cardiovascular outcomes, potentially capturing only early-onset events. Both limitations would result in non-differential misclassification or incomplete outcome ascertainment, biasing our estimates toward the null. Therefore, our findings should be interpreted as conservative, and the true long-term association may be stronger than observed. Second, this study population was restricted to women aged 18–55 years given the epidemiological characteristics of endometriosis. However, this restriction limits the ability to evaluate cardiovascular risk in the postmenopausal period. Hence, this study primarily examined whether endometriosis was associated with early-onset cardiovascular events, and our findings may underestimate the true long-term cardiovascular risk associated with endometriosis. Future studies with extended follow-up periods including women aged over 55 are needed to clarify the lifetime cardiovascular risk associated with endometriosis. Finally, information on several important cardiovascular risk factors, including smoking, obesity, dyslipidaemia, physical activity, alcohol consumption, and family history, was unavailable in the BMCDE database, and thus we could not adjust for these confounding variables. To partially mitigate this limitation, we used hypertension, diabetes mellitus, and the use of statins and aspirin intakes as proxy indicators of high cardiovascular risk.

## CONCLUSIONS

In summary, this population-based cohort study found that endometriosis was associated with an increased risk of CVD. Our findings suggest that women with endometriosis may benefit from CVD prevention to reduce their long-term cardiovascular risk and associated healthcare burden. Further prospective studies with extended follow-up and more comprehensive adjustment for cardiovascular risk factors are warranted to confirm these findings.

## Additional material


Online Supplementary Document

## Data Availability

**Data availability:** Data in the manuscript will be made available upon request and pend approval to the corresponding authors.
