# Supplementary material for: Association between endometriosis and cardiovascular disease: a population-based cohort study
Source: J Glob Health. 2026 Sep 18;16:04326. doi: 10.7189/jogh.16.04326 (PMC13587159; doi:10.7189/jogh.16.04326)
Supplement: Supplementary file 1 [file jogh-16-04326-s001.pdf]

Supplement to: Wang X, Xie S, Yu H, Wang K, Wang H, Yang R, Qin X, Wu, Y, Hu Y. Association between endometriosis and cardiovascular disease: a population-based cohort study. J Glob Health. 2026;16:04326.

Supplemental Table 1. Adherence to Journal of Global Health's GRABDROP guidelines items.

**JoGH guideline items**
1. Please list all papers published by each co-author in previous 3 years that were based on secondary analysis of a big data repository
[1] A R., Yu H., Wang K., Yang R., Wang H., Zheng L., Wu J., Wu Y. (2026). Aspirin use and risk of depression: a cross-sectional study. Frontiers in Pharmacology, 17. DOI: http://dx.doi.org/10.3389/fphar.2026.1721286.
[2] Yang R., Zheng Y., Yu H., Wu J., Wang S., Chen H., Wang M., Qin X., Wu T., Chang C., Wu Y., Hu Y. (2026). Impact of comprehensive tobacco control policies on stroke and acute myocardial infarction among patients with hypertension and type 2 diabetes in Beijing, China: An interrupted time-series analysis. Addiction (Abingdon, England), 121(1), 186-195. DOI: http://dx.doi.org/10.1111/add.70178.
[3] Yu H., Xu N., Yang R., Zheng L., Wang K., Wang H., Wu J., Xie S., Chen Y., Li T., Chang Z., Lv X., Hu Y., Yu X., Wu Y. (2026). Real-World treatment patterns in inpatients with newly diagnosed bipolar disorder in China: A descriptive longitudinal study. Journal of Affective Disorders, 410. DOI: http://dx.doi.org/10.1016/j.jad.2026.121966.
[4] Yang R., Wu J., Yu H., Wang S., Chen H., Wang M., Qin X., Wu T., Wu Y., Hu Y. (2024). Effect of statin therapy patterns on readmission and mortality in patients with intracerebral hemorrhage. Journal of Thrombosis and Thrombolysis, 57(1), 132-142. DOI: http://dx.doi.org/10.1007/s11239-023-02870-2.
[5] Wu J., Wu Y., Wu Y., Yang R., Yu H., Wen B., Wu T., Shang S., Hu Y. (2024). The impact of heat waves and cold spells on pneumonia risk: A nationwide study. Environmental Research, 245. DOI: http://dx.doi.org/10.1016/j.envres.2023.117958.
[6] Yang R., Yu H., Wu J., Wang S., Chen H., Wang M., Qin X., Wu T., Wu Y., Hu Y. (2024). Association of benzodiazepine and Z-hypnotic use with cardiovascular disease risk: insights from a prospective study of 10 million people in China. Psychiatry and Clinical Neurosciences, 78(12), 776-782. DOI: http://dx.doi.org/10.1111/pcn.13735.
[7] Yang R., Yu H., Wu J., Chen H., Wang M., Wang S., Qin X., Wu T., Wu Y., Hu Y. (2023). Metformin treatment and risk of diabetic peripheral neuropathy in patients with type 2 diabetes mellitus in Beijing, China. Frontiers in Endocrinology, 14. DOI: http://dx.doi.org/10.3389/fendo.2023.1082720.
[8] Zhao T., Zhang Y., Wang K., Yu H., Lin L., Qin X., Wu T., Chen D., Wu Y., Hu Y. (2023). Identifying Risk Factors for Aspiration in Patients Hospitalized with Community-Acquired Pneumonia. International Journal of Clinical Practice, 2023. DOI: http://dx.doi.org/10.1155/2023/2198259.
[9] Tian Y., Wu J., Wu Y., Wang M., Wang S., Yang R., Wang X., Wang J., Yu H., Li D., Wu T., Wei J., Hu Y. (2023). Short-term exposure to reduced specific-size ambient particulate matter increase the risk of cause-specific cardiovascular disease: A national-wide evidence from hospital admissions. Ecotoxicology and Environmental Safety, 263. DOI: http://dx.doi.org/10.1016/j.ecoenv.2023.115327.
[10] Tian Y., Wu J., Liu H., Wu Y., Si Y., Wang X., Wang M., Wu Y., Wang L., Li D., Wang W., Chen L., Wei C., Wu T., Gao P., Hu Y. (2023). Ambient temperature variability and hospital admissions for pneumonia: A nationwide study. Science of the Total Environment, 856. DOI: http://dx.doi.org/10.1016/j.scitotenv.2022.159294.

2. Please explain the key elements of your study design and the use of the available datasets that make your study an original scientific contribution
We conducted a historical, population-based matched cohort study using data from the Beijing Medical Claim Data for Employees (BMCDE) between 2010 and 2017. We identified women aged 18-55 years with a new diagnosis of endometriosis (ICD-10 code N80) between January 1, 2010 and December 31, 2017. All patients were matched to 4 women with no diagnosis of endometriosis by age (±1 year), and year of endometriosis diagnosis (±1 year) who were randomly selected from the BMCDE database. The incident CVD events were observed from the index date to the exit date. The exit date was the earliest of the outcome, study end date (December 31, 2017), or death. The primary outcome was the incident diagnosis of any CVD events (ICD-10 codes I00-I99). Secondary outcomes were major CVD (including acute myocardial infarction [ICD-10 code I21], ischemic heart disease [ICD-10 code I20-I25], and heart failure [ICD-10 code I50]), and cerebrovascular disease (including stroke [ICD-10 codes I60-I64] and other cerebrovascular diseases [ICD-10 codes I65-I69]). Covariates included hypertension, and diabetes mellitus. They were acquired at the index date. Hypertension was defined as having been diagnosed with an ICD-10 code of I10-I15 or having anti-hypertensive prescriptions. Diabet**es** mellitus was defined as having been diagnosed with an ICD-10 code of E11-E14 or having ≥1 prescription of hypoglycemic agents. Medications included progesterone, gonadotropin-releasing hormone (GnRH) agonists, statins, and aspirin.
3. Please list all publications that addressed similar research questions in the same dataset and indicate where you cited them in your paper
Wu Y, Wang Z, Zheng Y, Wang M, Wang S, Wang J, Wu J, Wu T, Chang C, Hu Y: The impact of comprehensive tobacco control policies on cardiovascular diseases in Beijing, China. Addiction (Abingdon, England) 2021, 116(8):2175-2184. This article has been cited in the 'Data source and study population' subsection of the Methods section.
4. Please explain how you addressed multiple testing through an appropriately rigorous statistical threshold and indicate this in the methods section
In this study, we prespecified one primary outcome and two secondary outcomes, and all analyses were conducted strictly in line with the prespecified hypotheses. Subgroup and mediation analyses were performed for exploratory purposes only. Importantly, no multiple testing of the same hypothesis beyond the stated outcomes. Given the confirmatory nature of the primary and secondary outcome analyses and the limited number of comparisons, we consider the risk of inflated type I error due to multiple testing to be minimal. We have included a clear description of the analytical plan and the prespecified nature of the outcome evaluations in the methods section.
5. Please declare to what extent have AI chatbots been used in developing your paper and to which parts of the paper did they contribute
Not applicable.

Supplemental Table 2. Hazard ratios for cardiovascular disease events stratified by age group

	Events, n	Crude HR (95% CI)	*P* value	Adjusted HR (95% CI)*	*P* value	*P* for interaction
**All CVD**						0.942
18-44 years	101	1.88 (1.23, 2.86)	0.003	1.51 (0.92-2.47)	0.102	
45-55 years	310	1.66 (1.30, 2.12)	<0.001	1.69 (1.31-2.18)	<0.001	
**Major CVD**						0.552
18-44 years	20	2.30 (0.90, 5.84)	0.081	2.43 (0.77-7.64)	0.128	
45-55 years	158	1.71 (1.22, 2.41)	0.002	1.81 (1.28-2.56)	<0.001	
**CBD**						0.211
18-44 years	58	1.40 (0.78, 2.51)	0.267	1.14 (0.58-2.22)	0.708	
45-55 years	134	1.67 (1.15, 2.42)	0.007	1.74 (1.16-2.60)	0.008	

Abbreviations: CBD=cerebrovascular disease; CVD=cardiovascular disease; HR=hazard ratio; CI=confidence interval.

*Adjusted for urbanization, hypertension and diabetes mellitus.

Supplemental Table 3. Mediation analysis to assess whether medications mediated the associations between endometriosis and cardiovascular disease risk

		Direct effect			Indirect effect			Total effect		Proportion mediated (%)	*P* value
		HR (95% CI)*	*P* value		HR (95% CI)*	*P* value		HR (95% CI)*	*P* value		
**All CVD**											
Statins	Overall	1.23 (0.99-1.52)	0.058		1.40 (1.34-1.47)	<0.001		1.72 (1.39-2.13)	<0.001	68.30	<0.001
	18-44 years	1.43 (0.94-2.19)	0.096		1.14 (1.07-1.21)	<0.001		1.63 (1.07-2.48)	0.023	30.99	0.013
	45-55 years	1.23 (0.96-1.58)	0.098		1.50 (1.43-1.58)	<0.001		1.85 (1.44-2.38)	<0.001	72.81	<0.001
Aspirin	Overall	1.16 (0.94-1.44)	0.174		1.49 (1.42-1.57)	<0.001		1.73 (1.40-2.14)	<0.001	78.15	<0.001
	18-44 years	1.34 (0.88-2.05)	0.177		1.21 (1.12-1.31)	<0.001		1.63 (1.07-2.48)	0.023	45.74	0.009
	45-55 years	1.18 (0.92-1.51)	0.190		1.58 (1.50-1.67)	<0.001		1.86 (1.45-2.39)	<0.001	79.16	<0.001
Progesterone	Overall	1.54 (1.24-1.92)	<0.001		1.03 (0.98-1.08)	0.179		1.59 (1.29-1.97)	<0.001	8.72	0.192
	18-44 years	1.55 (1.00-2.41)	0.051		1.02 (0.94-1.10)	0.670		1.58 (1.03-2.42)	0.036	4.82	0.674
	45-55 years	1.59 (1.24-2.06)	<0.001		1.03 (0.96-1.11)	0.407		1.64 (1.29-2.10)	<0.001	7.68	0.405
GnRH agonists	Overall	1.66 (1.33-2.06)	<0.001		0.94 (0.86-1.04)	0.250		1.57 (1.26-1.95)	<0.001	-16.12	0.295
	18-44 years	1.50 (0.95-2.36)	0.081		1.09 (0.86-1.39)	0.478		1.64 (1.06-2.52)	0.026	21.54	0.452
	45-55 years	1.69 (1.32-2.17)	<0.001		0.93 (0.85-1.03)	0.158		1.58 (1.23-2.03)	<0.001	-19.53	0.217
**Major CVD**											
Statins	Overall	1.09 (0.79-1.51)	0.583		1.67 (1.59-1.75)	<0.001		1.82 (1.32-2.52)	<0.001	88.53	<0.001
	18-44 years	1.18 (0.47-2.95)	0.730		1.68 (1.46-1.94)	<0.001		1.97 (0.78-5.00)	0.152	81.97	0.038
	45-55 years	1.17 (0.83-1.65)	0.371		1.64 (1.56-1.72)	<0.001		1.92 (1.36-2.71)	<0.001	81.46	<0.001
Aspirin	Overall	1.05 (0.76-1.44)	0.785		1.74 (1.65-1.84)	<0.001		1.82 (1.32-2.52)	<0.001	94.42	<0.001
	18-44 years	1.17 (0.47-2.93)	0.741		1.69 (1.42-2.02)	<0.001		1.97 (0.77-5.03)	0.154	82.80	0.038
	45-55 years	1.13 (0.80-1.60)	0.483		1.70 (1.61-1.79)	<0.001		1.92 (1.36-2.72)	<0.001	85.76	<0.001
Progesterone	Overall	1.55 (1.11-2.17)	0.010		1.04 (0.97-1.13)	0.264		1.62 (1.18-2.23)	0.003	11.03	0.282

	18-44 years	1.65 (0.62-4.40)	0.315		1.11 (0.86-1.42)	0.424		1.83 (0.72-4.67)	0.207	21.26	0.457
	45-55 years	1.64 (1.15-2.33)	0.006		1.02 (0.93-1.13)	0.632		1.68 (1.19-2.36)	0.003	5.91	0.630
GnRH agonists	Overall	1.64 (1.18-2.29)	0.004		1.00 (0.85-1.18)	0.977		1.64 (1.18-2.27)	0.003	-0.63	0.977
	18-44 years	1.56 (0.55-4.40)	0.404		1.29 (0.69-2.42)	0.425		2.01 (0.78-5.21)	0.151	44.86	0.370
	45-55 years	1.66 (1.17-2.36)	0.005		1.02 (0.85-1.21)	0.845		1.69 (1.19-2.40)	0.004	4.24	0.842
**CBD**											
Statins	Overall	1.22 (0.89-1.67)	0.220		1.33 (1.25-1.41)	<0.001		1.62 (1.18-2.22)	0.003	64.80	<0.001
	18-44 years	1.12 (0.62-2.02)	0.717		1.13 (1.04-1.21)	0.002		1.26 (0.70-2.27)	0.447	54.78	0.407
	45-55 years	1.31 (0.90-1.91)	0.153		1.46 (1.35-1.57)	<0.001		1.91 (1.32-2.78)	0.001	65.55	<0.001
Aspirin	Overall	1.15 (0.84-1.59)	0.373		1.41 (1.32-1.51)	<0.001		1.63 (1.19-2.23)	0.002	75.34	<0.001
	18-44 years	1.02 (0.56-1.85)	0.955		1.23 (1.10-1.36)	<0.001		1.25 (0.69-2.25)	0.461	93.09	0.416
	45-55 years	1.28 (0.88-1.86)	0.201		1.50 (1.38-1.62)	<0.001		1.92 (1.32-2.79)	0.001	69.68	<0.001
Progesterone	Overall	1.55 (1.12-2.14)	0.008		0.99 (0.93-1.05)	0.774		1.54 (1.12-2.11)	0.007	-2.47	0.775
	18-44 years	1.33 (0.73-2.44)	0.348		0.95 (0.89-1.01)	0.095		1.26 (0.70-2.29)	0.439	-26.18	0.431
	45-55 years	1.69 (1.15-2.48)	0.007		1.00 (0.90-1.11)	0.977		1.69 (1.17-2.45)	0.005	0.37	0.977
GnRH agonists	Overall	1.65 (1.20-2.26)	0.002		0.87 (0.78-0.96)	0.007		1.43 (1.04-1.96)	0.028	-50.99	0.094
	18-44 years	1.22 (0.65-2.29)	0.527		1.01 (0.73-1.38)	0.961		1.23 (0.67-2.26)	0.499	4.15	0.960
	45-55 years	1.82 (1.25-2.63)	0.002		0.84 (0.83-0.86)	<0.001		1.53 (1.06-2.22)	0.024	-53.34	0.006

Abbreviations: CBD=cerebrovascular disease; CVD=cardiovascular disease; HR=hazard ratio; CI=confidence interval.

*Adjusted for urbanization, hypertension and diabetes mellitus.

Note: Mediation proportions are reported only for descriptive purposes. Formal interpretation of the proportion mediated is warranted only when both the indirect effect and the total effect are statistically significant. Estimates exceeding 100% or negative values occur when the indirect and total effects are not consistently significant, and such estimates should not be interpreted as meaningful effect sizes.
